# Sequence-based Functional Metagenomics Reveals Novel Natural Diversity of Functional CopA in Environmental Microbiomes

**DOI:** 10.1016/j.gpb.2022.08.006

**Published:** 2022-09-08

**Authors:** Wenjun Li, Likun Wang, Xiaofang Li, Xin Zheng, Michael F. Cohen, Yong-Xin Liu

**Affiliations:** 1 Hebei Key Laboratory of Soil Ecology, Centre for Agricultural Resources Research, Institute of Genetics and Developmental Biology, Chinese Academy of Sciences, Shijiazhuang 050022, China; 2 University of Chinese Academy of Sciences, Beijing 100049, China; 3 Department of Biology, Sonoma State University, Rohnert Park, CA 94928, USA; 4 State Key Laboratory of Plant Genomics, Institute of Genetics and Developmental Biology, Chinese Academy of Sciences, Beijing 100101, China

**Keywords:** Functional metagenomics, Natural diversity, CopA, Evolutionary trace analysis, Cu resistance

## Abstract

Exploring the **natural diversity** of functional genes/proteins from environmental DNA in high throughput remains challenging. In this study, we developed a sequence-based **functional metagenomics** procedure for mining the diversity of copper (Cu) resistance gene ***copA*** in global microbiomes, by combining the metagenomic assembly technology, local BLAST, **evolutionary trace analysis** (ETA), chemical synthesis, and conventional functional genomics. In total, 87 metagenomes were collected from a public database and subjected to *copA* detection, resulting in 93,899 hits. Manual curation of 1214 hits of high confidence led to the retrieval of 517 unique CopA candidates, which were further subjected to ETA. Eventually, 175 novel *copA* sequences of high quality were discovered. Phylogenetic analysis showed that almost all these putative CopA proteins were distantly related to known CopA proteins, with 55 sequences from totally unknown species. Ten novel and three known *copA* genes were chemically synthesized for further functional genomic tests using the Cu-sensitive *Escherichia coli* (Δ*copA*). The growth test and Cu uptake determination showed that five novel clones had positive effects on host **Cu resistance** and uptake. One recombinant harboring *copA*-like 15 (*copAL15*) successfully restored Cu resistance of the host with a substantially enhanced Cu uptake. Two novel *copA* genes were fused with the *gfp* gene and expressed in *E. coli* for microscopic observation. Imaging results showed that they were successfully expressed and their proteins were localized to the membrane. The results here greatly expand the diversity of known CopA proteins, and the sequence-based procedure developed overcomes biases in length, screening methods, and abundance of conventional functional metagenomics.

## Introduction

Knowledge on protein natural diversity is important for both evolutionary and bioengineering studies. The natural diversity of genes/proteins like the DNA-directed RNA polymerase subunit beta (RpoB) [1] and the nitrogenase iron protein (NifH) [2] is widely utilized in microbial phylogenetics, particularly for identifying and describing the nonculturable ‘dark matter’ [3]. The known present-day functional proteins represent a small fraction of the proteins that have arisen over the millions or billions of years of natural selection [4]. High-throughput recovery of the natural diversity of functional protein variants may pave a way to the quest of how the existing natural proteins differ from random sequences [5], and to protein engineering based on the large-scale library of sequence variants of natural selection instead of directed mutagenesis. Sequence-based enzyme redesign has been shown to be successful in the discovery of esterase and endopeptidase of enhanced activity [6].

For functional genes other than phylo-marker genes, the detection of homologous genes/proteins traditionally relies on the genomics exploration of a pure culture. Expansion of full-genome sequencing greatly enhances our ability to assess the natural diversity of a functional gene/protein, whereas for some genes/proteins with non-ubiquitous cellular functions like metal resistance [7], probing their natural diversity remains difficult due to their low abundance in the environment and the lack of characterized sequences in common databases [8]. Metagenomes contain the full genetic information of environmental DNA (eDNA) and provide an ideal approach to exploring the natural diversity of functional genes/proteins. Mining functional genes from the metagenomes includes the function-based and sequence-based approaches [9]. Function-based screening leads to the discovery of novel antibiotic resistance genes [10], [11], biosurfactants [12], and a variety of biocatalysts [13], [14]. Sequence-based functional metagenomics bypasses the limitations of the function-based approaches in the availability of screening methods and redundant isolation [15]. Unfortunately, for many genes, particularly those of large size, such as metal transporter genes, it remains challenging to recover full-length genes from eDNA in a high-throughput manner due to difficulties in polymerase chain reaction (PCR) detection, degenerated primer design, or the availability of known homologs [16].

The core gene for microbial copper (Cu) resistance, *copA*, is such a gene of large size of around 2000 bp. It normally possesses a low abundance in natural eDNA, and has a limited number of characterized variants to date. Reports on Cu resistance genetic determinants can be traced back to decades ago. Tetaz and Luke reported that plasmid pRJ1104 carried by *Escherichia coli* K-12 conferred enhanced Cu resistance [17]. The Cu-resistant operon *cop* was first found in *Enterococcus hirae*, which contains regulator genes *copY* and *copZ* encoding a repressor and a chaperone, respectively, as well as the structural genes *copA* and *copB* that mediate Cu transport [18].

CopA is one of the most well-known microbial metal transporters [19]. Studies have shown that CopA was able to protect *Streptococcus suis* through Cu efflux [20]. The lack of CopA can make *E. coli* sensitive to Cu and result in the accumulation of Cu^+^ in cells [21]. Regularly, each CopA monomer from *E. coli* binds two Cu^+^ and subsequently transfers them to a periplasmic Cu chaperone (CusF) coupled to ATP hydrolysis, thus resulting in the transport of Cu^+^ from cytoplasm to periplasm through the CusCBA trimer protein complex [22]. It is worth noting that the role of CopA has been reported to be distinct in different bacteria. For instance, in *E*. *hirae*, CopA was annotated as a Cu importer [23], whereas the *Bacillus subtilis* CopA was found to be a Cu exporter [24], [25]. Exploration of more functional CopA proteins from the tremendous reservoir of eDNA may not only facilitate the sequence-based protein design of metal transporters, but also expand the functional diversity of known CopA proteins.

Homology-based annotation has predicted a large number of novel CopA proteins from eDNA of Cu-contaminated environments, which remains to be verified both bioinformatically and experimentally [26], [27], [28]. A pipeline of sequence-based functional metagenomics has been developed, and the high-throughput retrieval of metallothionein genes, a family of short genes of around 70 bp encoding Cys-rich metal-binding proteins, from a soil microbiome was realized [8]. Similarly, this procedure was applied in the current study to explore CopA, a much longer metal transporter, from eDNA. To achieve this goal, metagenomes from various environmental microbiomes worldwide were collected from a public database metagenomics rapid annotation using subsystem technology (MG-RAST) server [29], and subjected to the retrieval of full-length *copA* and functional analysis. Evolutionary trace analysis (ETA) was carried out using a cluster of experimentally-tested CopA proteins to generate sequence features for evaluating the CopA candidates. Ten candidate *copA* genes were randomly selected based on the phylogenetic analysis and chemically synthesized for subsequent heterologous expression in Cu-sensitive *E. coli* JW0473-3 (Δ*copA*). Cu uptake by Cu-sensitive *E. coli* harboring the synthesized *copA* genes was determined. Two clones of *copA* were fused with the green fluorescent protein gene (*gfp*) tag and visualized. Overall, the results here demonstrate the power of sequence-based functional metagenomics in mining or even exhausting the natural diversity of a functional gene in microbiomes. The candidate *copA* genes detected here may have a distinct mechanism for conferring host Cu resistance.

## Results

### ETA and structure characteristics of known CopA proteins

**Figure 1:**
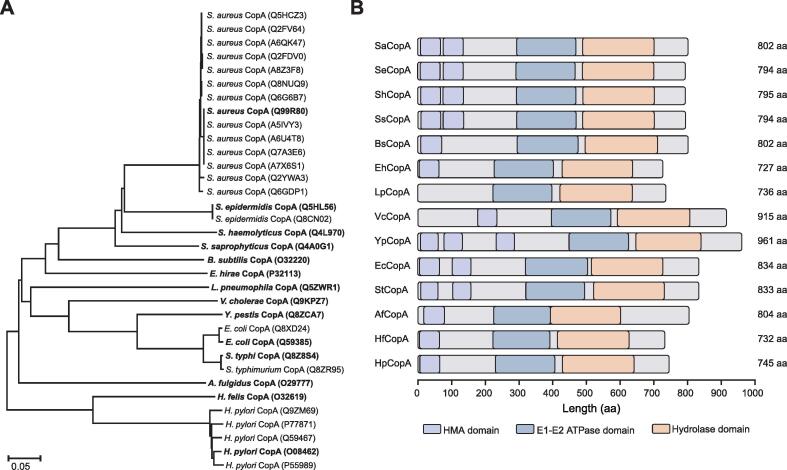
**ETA and structural features ofkonwnCopA proteins** **A.** ETA of 34 known CopA proteins. Amino acid sequences of the proteins were used to create a phylogenetic tree using MEGA 7.0. The proteins with the bolded names were used for further structural analysis in (B). **B.** Functional domains within the 14 groups of known CopA proteins. Sequences of the 34 known CopA proteins were merged into 14 groups according to species source. Numbers on the right represent the length of the protein sequences. ETA, evolutionary trace analysis; HMA, heavy metal transporting ATPase; aa, amino acid.

Thirty-four CopA proteins have been reported in UniPort, and phylogenetic analysis (Figure 1A) in this study indicated that the 34 CopA proteins were mainly from 14 bacterial species, among which 14 closely related CopA proteins were found to be derived from *Staphylococcus aureus* and another five were from *Helcobacter pylori* (Table S1), suggesting that there may be many undiscovered CopA proteins out there. All known CopA proteins function in Cu efflux, except for the CopA from *E. hirae*, which was annotated as copper-importing P-type ATPase. In terms of protein length, CopA generally contains about 800 amino acids with the longest one (961 amino acids) from *Yersinia pestis* (Figure 1B). The number of heavy metal transporting ATPase (HMA) domains of the 14 groups of CopA ranges from 1 to 3, except for CopA from *Legionella pneumophila* subsp*. pneumophila* with no HMA domain found (Figure 1B). All the CopA proteins possess an E1-E2 ATPase domain (Figure 1B), which is a Cu binding and efflux structure related to ATP hydrolysis and realizing Cu binding and efflux through conformational variation [30].

### Diverse and novel *copA* genes were detected from the global microbiomes

Of the 88 metagenomic datasets, 47 of them had their sequences undergone pre-processing/quality control to ensure their quality. The assemblage quality varied among the metagenomic datasets, largely due to that the data retrieved from MG-RAST differed in sequencing methods, thus resulting in differences in data size and sequence length (Table S2). One low-quality dataset mgm4754648 was eliminated from the library and the assembly results of the rest 46 metagenomes were included. Eventually, 5,500,798 contigs from the assemblage and 134,409,173 amino acid sequences from other 41 datasets were input for local blast. In total, 87 databases were subjected to subsequent analysis.

A total of 93,899 hits were obtained after searching the metagenomic assemblages against the known-CopA database. Then 1214 returned records of high quality were selected for manual retrieval of CopA candidates from the hits of the highest confidence. Through ORF-finder analysis, 517 sequences with length ranging from 500 to 900 amino acids were preserved and subjected to transmembrane helix prediction. As a result, predicted by TMHMM and Pfam, 315 of them possessed transmembrane helices. Among the 315 sequences, 222 contained metal transport-related ATPase domains (HMA and E1-E2 ATPase). By manual curation of the 222 sequences on their CXXC, HXXH, or CXC amino acid conservative domains, 175 *copA*-like genes were retrieved.

Taxonomy of the 175 *copA*-like genes was classified by Kraken 2 (see Material and methods; Table S3). They were found to be mainly distributed in five phyla: Proteobacteria, Actinobacteria, Euryarchaeota, Bacteroidetes, and Firmicutes (Figure S1A). Among them, 120 sequences belonged to 74 known species, 69 genera, and 47 families (Figure S1A). Other 55 sequences were annotated as unknown species (Table S3). At the genus level, 98.6% of the *copA*-like genes were affiliated to 68 novel genera in this study, with only one genus having known *copA* genes (Figure S1B). At the species level, all the 74 species were first-time reported to harbor putative *copA* genes (Figure S1C). These novel *copA* genes greatly extend the taxonomic diversity of known *copA* genes.

**Figure 2:**
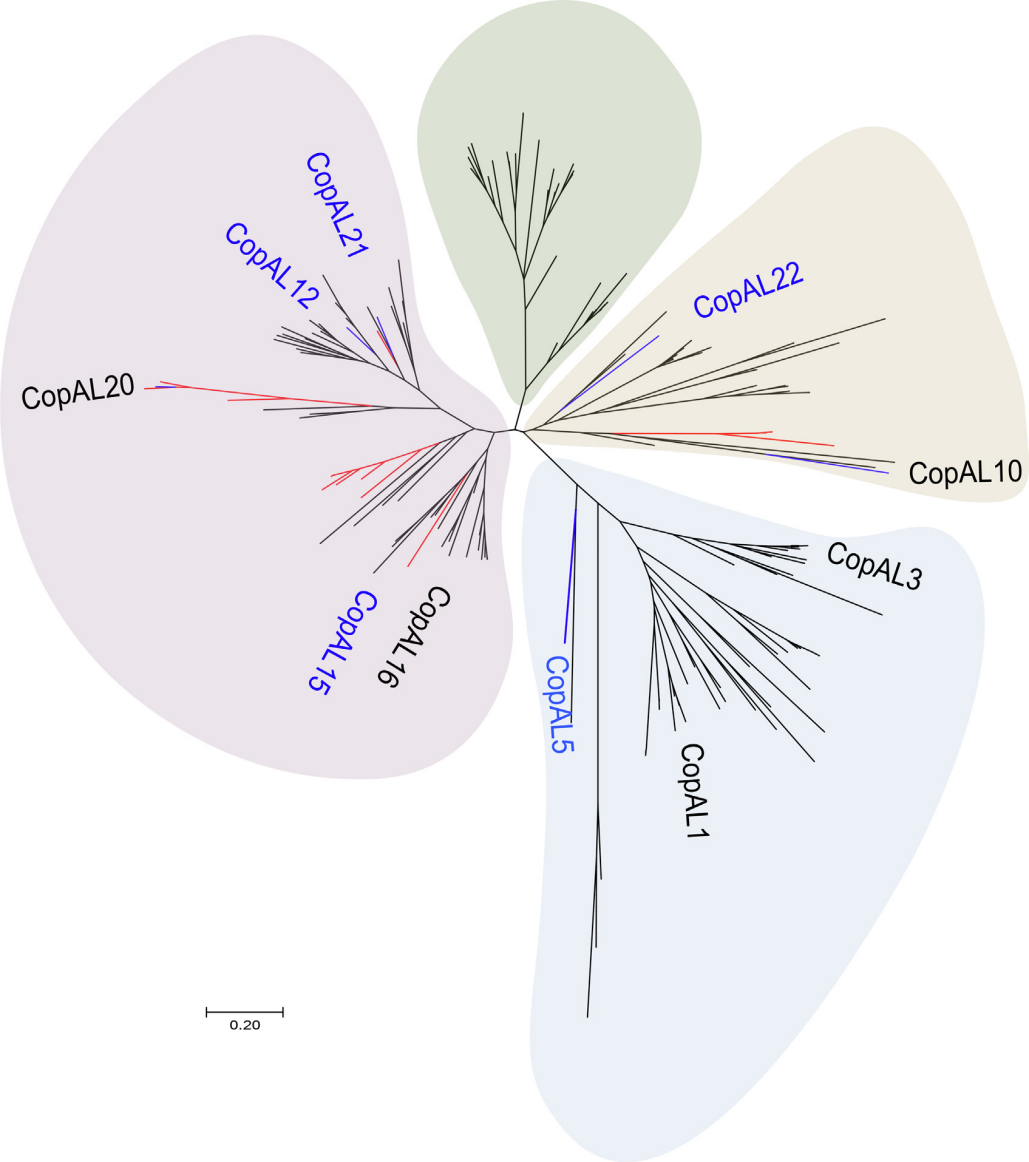
**ETA of the 175CopA-likeand34 known CopA proteins** Lines in red represent the 34 known CopA proteins, and lines in blue represent the 10 CopA-like proteins that were functionally tested via the experiments detailed herein. CopA-like (CopAL) names in blue are the 5 clones that altered the Cu resistance capacity of the host *Escherichia coli* JW0473-3 (Δ*copA*) relative to the negative control. The phylogenetic tree of the 175 CopA-like proteins as well as the 34 known CopA proteins was constructed with MEGA 7.0 using the maximum likelihood method and 1000 bootstrap replicates.

ETA results of the 175 CopA-like (CopAL) proteins and the 34 known CopA proteins revealed that the sequences were separated into four main branches; however, the 34 known CopA proteins were only distributed in two of the branches. A large proportion of the CopA-like proteins were located in different developmental branches from the known CopA proteins (Figure 2).

### Selected ***copA***-like genes resulted in intriguing resistance in host cells

**Figure 3:**
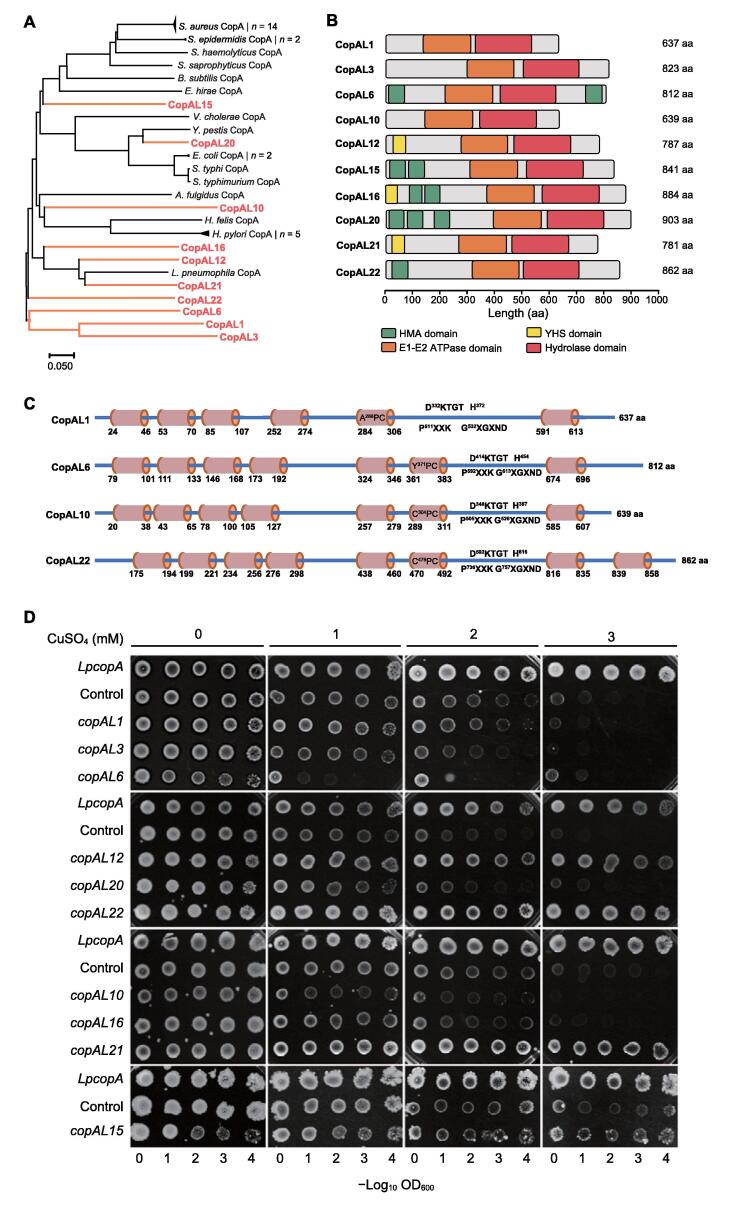
**Functional genomic verification of the 10 selected *copA*-likegenes** **A.** ETA of the 34 known CopA proteins and the 10 CopA-like proteins. Amino acid sequences were used to construct a phylogenetic tree using MEGA 7.0. **B.** Functional domains within the 10 selected CopA-like proteins. **C.** Schematic illustration of the average polypeptide composition of the 10 selected CopA-like proteins. Among them, CopAL1 represents the average composition of both CopAL1 and CopAL3; CopAL22 represents the average composition of CopAL12, CopAL15, CopAL16, CopAL20, CopAL21, and CopAL22. **D.** Drop assay of *E. coli* JW0473-3 (Δ*copA*) strains harboring each of the 10 *copA*-like genes. *LpcopA* indicates the *E. coli* JW0473-3 (Δ*copA*) strain harboring recombinant pTR-*LpcopA* (positive control); control indicates the *E. coli* JW0473-3 (Δ*copA*) strain harboring the empty pTR vector (negative control). YHS, tyrosine–histidine–serine.

Ten *copA*-like genes were selected for chemical synthesis. Amino acid sequences of the 10 selected *copA*-like genes were back compared with the 34 CopA sequences in the local database by phylogenetic analysis (Figure 3A). Overall, sequences of CopAL15, CopAL20, CopAL10, CopAL16, CopAL12, and CopAL21 were closer to known CopA proteins in the local database, whereas sequences of CopAL22, CopAL6, CopAL1, and CopAL3 were divergent from the known ones and presented independent branches in the phylogenetic tree (Figure 3A). More specifically, CopAL6, CopAL1, and CopAL3 were similar to each other, and they were separated from CopA22. In the phylogenetic tree, sequences of CopAL16, CopAL12, and CopAL21 were similar to each other, and they possessed high homology with the CopA of *L. pneumophila* (Figure 3A). In addition, the sequence of CopAL15 was almost identical to the CopA proteins found in *Staphylococcus* spp., *B. subtilis*, and *E. hirae*. Notably, the CopA in *E. hirae* was the only one annotated as functioning in Cu import instead of efflux (Figure 3A).

The length of the 10 CopA-like proteins ranged from 637 to 903 amino acids. All 10 proteins contain one E1-E2 ATPase domain and one hydrolase domain, whereas the number of HMAs is different among them (Figure 3B). The location of the two HMAs in CopAL6 is different from that in other CopA-like proteins, which are found at the two ends of the sequence (Figure 3B). Additionally, tyrosine–histidine–serine (YHS) domain that can bind to transition-metal is predicted in CopAL12, CopAL16, and CopAL21 (Figure 3B). Notably, the protein sequences of CopAL12 and CopAL16 are highly similar to the known LpCopA that lacks an HMA domain.

CopAL12, CopAL15, CopAL16, CopAL20, CopAL21, and CopAL22 contain 8 transmembrane domains with a cysteine–proline–cysteine (CPC) trimer located within the sixth transmembrane domain (Figure 3C). CopAL10 has 7 transmembrane domains, and a CPC trimer is also located in the sixth transmembrane domain (Figure 3C). The transmembrane domain prediction of CopAL6 also showed 7 transmembrane domains, whereas the metal binding site of the sixth transmembrane domain is tyrosine–proline–cysteine (YPC) trimer. CopAL1 and CopAL3 only possess 6 transmembrane domains, with their fifth transmembrane domains containing alanine–proline–cysteine (APC) and serine–proline–cysteine (SPC) trimers, respectively (Figure 3C). Furthermore, all the 10 synthetic genes encode proteins which possess ATP binding sites, such as monohistidine (H) and aspartate–lysine–threonine–glycine–threonine (DKTGT) pentamer.

Ten *copA*-like genes were transformed into *E. coli* JW0473-3 (Δ*copA*) through a pTR vector. Growth of the *E. coli* JW0473-3 (Δ*copA*) strian transformed with the empty pTR vector (the negative control) was inhibited in 2 mM Cu solid medium and completely suppressed under 3 mM Cu stress, whereas growth was not restricted even in 3 mM Cu solid medium for the *E. coli* JW0473-3 (Δ*copA*) strian harboring *LpcopA* (the positive control) (Figure 3D). Accordingly, the function of the 10 *copA*-like genes was classified into three categories: (1) reduced Cu resistance of the host (*copAL6*); (2) enhanced Cu resistance of the host (*copAL12*, *copAL15*, *copAL21*, and *copAL22*); (3) no change in Cu resistance of the host (*copAL1*, *copAL3*, *copAL10*, *copAL16*, and *copAL20*). Additionally, Cu-sensitive strains harboring *copAL12*, *copAL15*, *copAL21*, and *copAL22* showed Cu resistance similar to that of the positive control (Figure 3D).

### Function verification of selected ***copA***-like genes in Cu-sensitive strain

**Figure 4:**
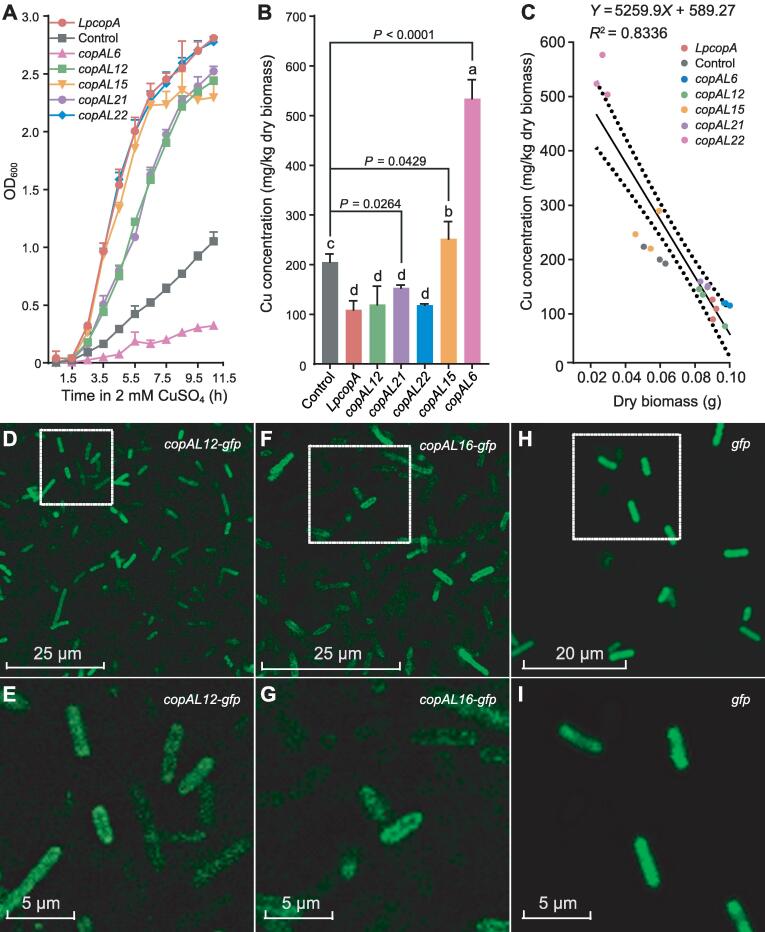
**Function verification of selected *copA*-like genes in Cu-sensitive strain** **A.** Growth of *E. coli* JW0473-3 (Δ *copA*) strains harboring *copAL6*, *copAL12*, *copAL15*, *copAL21*, *copAL22* along with the positive and negative controls determined in the liquid medium containging 2 mM CuSO_4_. **B.** Cu accumulation in *E. coli* JW0473-3 (Δ*copA*) harboring recombinant pTR plasmids, determined using ICP-MS under 1 mM CuSO_4_. **C.** Correlation analysis of biomass and Cu accumulation value. *LpcopA* indicates the *E. coli* JW0473-3 (Δ*copA*) strain harboring the recombinant pTR-*LpcopA* (positive control); control indicates the *E. coli* JW0473-3 (Δ*copA*) strain harboring the empty pTR vector (negative control). **D.** Green fluorescence of *E. coli* DH5α strain harboring a *copAL12*-*gfp* fusion. **E.** Magnified view of the image inside the white square in (D). The green fluorescence was observed only on the cell membrane. **F.** Green fluorescence of *E. coli* DH5α strain harboring a *copAL16*-*gfp* fusion. **G.** Magnified view of the image inside the white square in (F). The green fluorescence was observed only on the cell membrane. **H.** Green fluorescence of *E. coli* DH5α strain harboring *gfp*. **I.** Magnified view of the image inside the white square in (H). The green fluorescence was observed throughout the whole cell. Green fluorescence was visualized through a confocal laser scanning microscope. ICP-MS, inductively coupled plasma mass spectrometry.

Growth curves of the *E. coli* JW0473-3 (Δ*copA*) strains harboring *copAL6*, *copAL12*, *copAL15*, *copAL21*, and *copAL22* along with the positive and negative controls were determined in 2 mM Cu liquid medium (Figure 4A). All the five samples reached the stationary stage after 7-h incubation. Among them, the growth curves of Cu-sensitive strains harboring *copAL12*, *copAL15, copAL21,* and *copAL22* were similar to that of the positive control, and all of them grew faster than the negative control, whereas the growth of *E. coli* JW0473-3 (Δ*copA*) strain harboring *copAL6* was slower than the negative control, indicating that *copAL6* inhibits the growth of the sensitive host.

Under 1 mM Cu, sensitive strains harboring *copAL6* and *copAL15* had strong Cu uptake capacities, with a significantly higher bio-accumulation. Notably, under this Cu stress condition, bioaccumulation of Cu by *E. coli* JW0473-3 (Δ*copA*) strain harboring *copAL6* was 534.5 μg/g, which was the highest among all of the *copA*-like genes (Figure 4B). In contrast, Cu accumulation by *E. coli* JW0473-3 (Δ*copA*) strains harboring *copAL12*, *copAL21*, *copAL22*, and *LpcoA* (the positive control) were not significantly different from each other, yet they were all significantly lower than the negative control [Fisher’s least significant difference (LSD), *P* ≤ 0.05] (Figure 4B).

A correlation curve was created by plotting the biomass of transformants against Cu accumulation values (Figure 4C). A negative correlation was observed between Cu absorption and dry weight with a correlation coefficient (*R*^2^) of 0.8336. Among the Cu-sensitive strains harboring the five selected *copA*-like genes, the strain harboring *copAL6* showed the strongest Cu absorption capacity and relatively low biomass (Figure 4C). Interestingly, both Cu absorption capacity and harvested biomass of the Cu-sensitive strain harboring *copAL15* were significantly higher than the negative control (Fisher’s LSD, *P* ≤ 0.05) (Figure 4C).

Green fluorescence was observed in *E. coli* DH5α strains harboring *copAL12-gfp* and *copAL16-gfp* as well as in the positive control (Figure 4D, F, and H). In addition, strong fluorescence in both recombinants harboring *copAL12-gfp* and *copAL16-gfp* was observed to be localized to the cell membrane, suggesting that they may encode transmembrane transport proteins (Figure 4E, G, and I).

## Discussion

In the current study, a sequence-based functional metagenomics procedure was developed to mine the natural diversity of novel CopA proteins from eDNA. The procedure integrated metagenomic mining, ETA, chemical synthesis, and conventional functional genomics using a Cu-sensitive strain (Figure S2). The application of this procedure to explore the 87 metagenomes worldwide resulted in the discovery of 175 candidate *copA* genes of high confidence, among which 10 were randomly selected and chemically synthesized for functional genomic tests. Drop assays and growth curve determination showed that five *copA*-like genes altered the Cu resistance capacity of the host *E. coli* JW0473-3 (Δ*copA*) relative to the negative control, among which four (*copAL12*, *copAL15*, *copAL21*, and *copAL22*) restored Cu resistance of the sensitive strain and one (*copAL6*) reduced the Cu resistance of the host. Interestingly, these five *copA*-like genes exerted different impacts on Cu accumulation of the host in a manner that was significantly negatively correlated with the dry biomass of the host. Imaging evidence showed that *copAL12* fused with *gfp* was successfully expressed in host cells and its protein product was probably located in the cell membrane.

CopA belongs to the P_1B-2_ type ATPase family, one of the most well-known metal transport families. Evolutionary analysis of known CopA proteins revealed the conserved metal binding motifs on both termini [31]. Together with the recent reports on the complete or partial crystal structure of CopA proteins [32], [33], [34], [35], this enables the homology-based annotation of novel CopA proteins. It is estimated that *copA* abundance in the metagenomes was lower than 0.067%, which is very close to that of natural soil and much lower than Cu-contaminated mine wastes [26]. Although annotation of genes in metagenomes has reached a high level of sophistication, their function verification, particularly in the high-throughput fashion, is still difficult [36]. In this study, we randomly synthesized and tested ten full-length *copA*-like genes from the metagenomes, and to our surprise, five of these modified host Cu resistance and uptake of a Cu-sensitive *E. coli* strain. As a transporter is of large-size relative to other families, the heterologous expression of *copA* is not trivial. The successful detection of five putative functional *copA* genes indicates the high reliability and high possibility of the sequence-based procedure developed here, and we also expect that a large number of functional *copA* genes may present among the 175 *copA* candidates. With the lowering of the cost of DNA synthesis, we will gain the ability to apply this method to exhaustively assess the activity of these candidate *copA* genes and also explore the loss-of-function mutations.

Traditionally, screening target genes from eDNA with functional metagenomics approaches involves pressure selection [37]. However, this method is often problematic due to some experimental difficulties, particularly the bias in the length of the inserts [38]. Our previous study explored the possibility of using conventional functional metagenomics to detect novel Cu resistance genes from eDNA, whereas the results showed that all clones of Cu resistance were not transporter-like genes and with length shorter than 1.8 kb [37]. In contrast, metagenomics provides means of assessing the total genetic pool of all the microorganisms in a particular environment, which makes it possible to search for large-size functional genes, such as *copA*, without any biases [26], [31]. By means of the new metagenomics pipeline used in the present study, small DNA fragments were assembled into large-size contigs which could cover the whole *copA* sequence length of *ca.* 2400 bp. Again, this study demonstrates the power of sequence-based functional metagenomics in mining large-size functional genes which is difficult for traditional library-based metagenomics.

As mentioned above, although CopA proteins were annotated as Cu efflux proteins in most of the Cu-resistant microorganisms, one was found to function in Cu import in *E. hirae* [23]. Traditional gene mining generally involves obtaining pure cultures of the potential functional microbes first, and this may lead to a preference for Cu efflux-type CopA proteins. The procedure used in our study does not rely on the screening of Cu resistance in detecting candidate CopA proteins, and thus overcomes the bias for efflux functions. Accordingly, in our results, we found a novel CopA protein with a function of Cu import thereby increasing the Cu sensitivity of the host. Considering that all known Cu resistance systems like Cop, Pco, and Cus are of low abundance in natural environments [39], [40], the use of traditional metagenomics that relies on PCR cloning and library construction is not a realistic means for probing the natural diversity of these Cu resistance genes.

Although heterologous expression of targeted genes in a host can be extremely challenging, we achieved a relatively high success rate using a domesticated *E. coli* host strain [41]. Growth test and metal uptake determination showed successful detection of five candidate *copA* genes, demonstrating a 50% rate of detecting positive clones from the eDNA. In addition to the physiological evidence, imaging results based on GFP-fusion visualization further confirmed the successful expression of CopAL12 in the host and revealed its possible cell membrane localization. Although a candidate CopA, CopAL16, was also successfully expressed in the host and showed possible cell membrane localization based on the GFP-fusion visualization, it did not alter host Cu resistance (Figure 4F and G). In some cases, foreign DNA can be expressed in the heterologous host, but the gene function can be silent due to the lack of chaperones required for proper protein folding [9]. A protein that did not display antimicrobial activity in *E. coli* host did confer this activity to a *Ralston metallidurans* host [42], indicating the importance of using additional heterologous hosts to identify active clones that fail to express in the standard *E. coli* host [43]. We thus anticipate that the procedure developed here may be able to probe with a high rate of success the natural diversity of CopA and other proteins involved in metal transport.

Different from our previous study on metallothionein (MT) [8], *copA* is a gene with length ten times more than the *MT* genes, which makes it much more difficult for both successfully homologous detection and heterologous expression for function verification. Our recent study has explored the conventional functional genomics method for detecting *copA* from metagenomes, where none of the detected clones has a length more than 2000 bp [37]. Also, here we used a Δ*copA* mutant instead of the common *E. coli* strain used for *MT* genes to specifically verify the function of candidate *copA* genes. Such experimental evidence can be more convincing than conventional functional genomics. In all, this study provides a pipeline that is specifically for CopA mining in a high-throughput fashion, breaking through the length limitation of peptides mined from metagenomic data. Novel functional CopA sequences were detected which may be useful in bioengineering for bioremediation and the evolutionary exploration of metal resistance genes.

## Materials and methods

### Environmental metagenome collection and assembly

**Figure 5:**
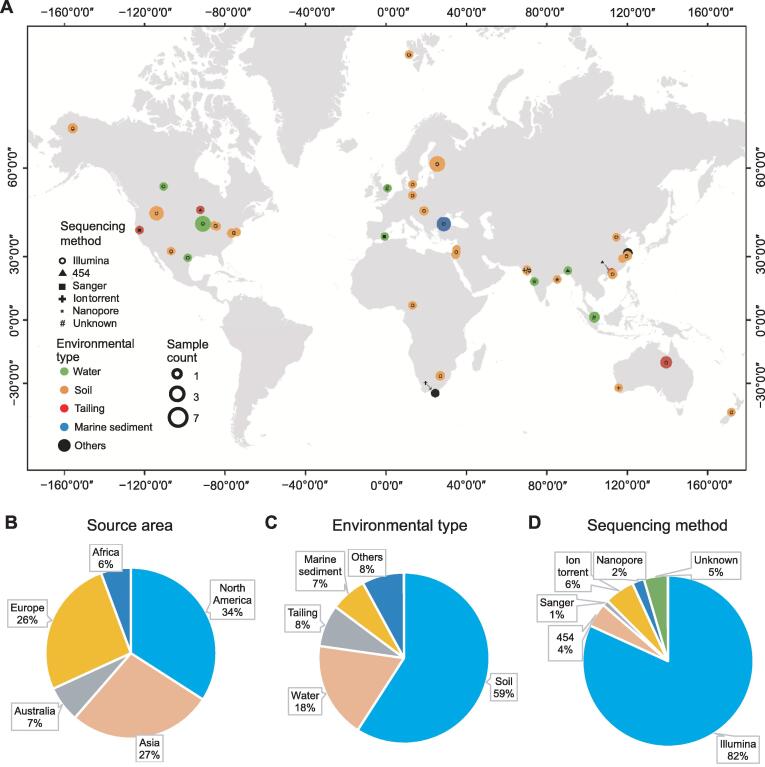
**Basic information of the 87 metagenomic datasets** **A.** Geographic distribution, environmental type, sample count, and sequencing method of the 87 datasets. **B.** Proportion of datasets among continents. **C.** Proportion of datasets of different environmental types. **D.** Proportion of datasets generated by different sequencing methods.

Eighty-eight environmental metagenomes and related metadata were collected from the MG-RAST [29]. The metagenomic datasets represented eDNA from a global diversity of habitats including farmland soil, forest soil, wastewater, contaminated soil, mine drainage, mine tailings, and ocean water (Figure 5). Among the datasets, 47 of them were quality-controlled DNA sequences, and host DNA was already removed (Table S2), thus data were assembled using the assembly module in Metawrap (v1.2.1) [44] through their in-house scripts (https://github.com/ebg-lab/CopA). One low-quality dataset mgm4754648 was removed from the study. The other 41 datasets were amino acid sequences, thus allowing for direct similarity comparison through BLASTP. Total base count, total read count, number of contigs, the largest contig length, and N50 were recorded (Table S2). Additionally, in order to facilitate the use of this method by other researchers, the detailed step-by-step protocol is provided in File S1.

### Known-CopA database construction for local BLAST

Thirty-four amino acid sequences marked with ‘manually annotated’ were retrieved from the Uniprot database (https://www.uniprot.org/) with ‘CopA’ as an entry. These sequences were experimentally characterized for either protein structure or metal-resistance function. The 34 sequences were mainly from 14 microorganisms, which are listed in Table S1. Data were re-formatted using makeblastdb from BLAST (v2.2.31; parameters ‘-in nucleotide.fa -dbtype nucl’ for nucleotide, and “-in protein.fa -dbtype prot” for amino acid) to create an index of database. The phylogenetic relationship of the 34 CopA sequences was constructed with MEGA 7.0 [45] using the maximum likelihood method and 1000 bootstrap replicates. Multiple sequence alignment was performed using ClustalW [46], and p-distance was calculated. All positions with less than 50% site coverage (namely ≥ 50% alignment gaps, missing data, or fuzzy bases) were eliminated. Domain and motif analyses of the 34 CopA sequences were followed by the same procedure as for the candidate CopA proteins below.

### Local BLAST for candidate *copA* detection

The environmental metagenomes were searched against the CopA database via local BLAST [47], which was done using a Linux system computer equipped with dual-core 2.2 × 2 GHz CPU and 192 G RAM. Briefly, nucleotide sequences with length greater than 2000 bp in the 47 assembled metagenomic datasets and amino acid sequences with length greater than 700 amino acids in the other 41 datasets were aligned against the CopA local database using the BLASTX and BLASTP alignment modules, respectively. Only those matches having an E value ≤ 1E−6 were recorded.

The matches obtained from BLAST were subjected to search for open reading frames (ORFs) using ORF finder (https://www.ncbi.nlm.nih.gov/orffinder/) with ATG as the initiation codon. Candidate ORFs encoding proteins with length ranging from 500 to 900 amino acids were selected and subjected to the transmembrane helix prediction using the TMHMM (https://www.cbs.dtu.dk/services/TMHMM-2.0/) online analysis platform. Functional domains were then predicted using Pfam (https://pfam.xfam.org/) with an E value ≤ 1E−6, and sequences without metal transporting domains were eliminated. Eventually, high-confidence *copA*-like sequences which encode proteins with CXXC, HXXH, or CXC conserved metal-binding domains were manually retrieved. A phylogenetic tree showing the evolutional relationship of the candidate CopA proteins was constructed using the aforementioned method.

### Bacterial strains and cultural conditions for function verification

**Supplementary Figure S3:**
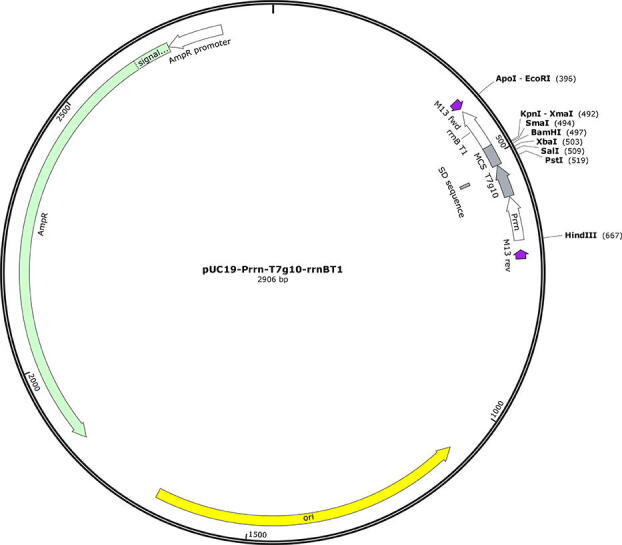
**A graphic map of *Escherichia coli* expression vector pUC19-Prrn-T7g10-rrnBT1 (PTR)**

A pTR vector carrying endonuclease sites *Pst*I and *Kpn*I was used as an expression vector in this study (Figure S3) [8], and Cu-sensitive *E. coli* JW0473-3 (Δ*copA*) was used as the host. *E. coli* JW0473-3 harboring the empty pTR vector was set as the negative control, and the one harboring recombinant pTR-*LpcopA* was used as the positive control. Common *E. coli* strain DH5α [F^–^ φ80*lacZ*ΔM15 Δ(*lacZYA*-*argF*)*U169 recA1 endA1 hsdR17*(r_k_^–^, m_k_^+^) *phoA supE44 thi-1 gyrA96 relA1* λ^–^; Catalog No. EC0112, Thermo Fisher Scientific, Carlsbad, CA] was used to store the recombinant plasmids and for heterologous expression of *gfp*-fused genes and imaging. Green fluorescence was observed through a confocal laser scanning microscope (TCS-SP8 Microsystems, Leica, Weztlar, Germany).

Luria-Bertani (LB) medium supplied with 100 mg/ml ampicillin and 50 mg/ml kanamycin was used to select *E. coli* JW0473-3 (Δ*copA*) recombinants.

### Taxonomy classification, comparison, and visualization

Taxonomic classification of the novel *copA* genes was performed using Kraken (v2.1.1) [48] based on the NCBI taxonomy database (v20210120). Then, the NCBI taxonomy IDs were converted into standard 7-rank table by Taxonkit (v0.7.0) [49]. The formatted taxonomic information is shown in Table S3. The data processing for Cladograms followed the guide of EasyAmplicon (v1.14) [50], and was finally visualized by ImageGP webserver (v1.0) [51] by calling GraPhlAn (v0.9.7) [52]. The comparison between known and novel taxonomies of *copA* genes was analyzed in EVenn webserver [53].

### Sequence synthesis

According to the phylogenetic analysis result of the 34 known CopA and 175 CopA-like proteins, 10 nucleotide sequences with a length of 2000–2700 bp were randomly selected from the *copA*-like genes and subjected to artificial DNA synthesis as well as subsequent function verification. Codon preference of the host, secondary structure of mRNA, and GC content were considered [54] for DNA synthesis to improve the expression efficiency in the host *E. coli*. The recombinant pTR-*copA* vectors were re-digested with *Pst*Ι and *Kpn*Ι restriction enzymes to examine the successfulness of insertion, and *copA*-like sequences were double-checked by sequencing on Sanger platform. pTR vectors carrying *gfp*-fused *copA*-like sequences were transformed into common *E. coli* DH5α for visualization of green fluorescence.

A phylogenetic tree of the 10 CopA-like and 34 known CopA sequences was created using MEGA 7.0 with the maximum likelihood method and 1000 bootstrap replicates.

### Drop assay for Cu resistance screening

The function of the 10 *copA*-like genes were verified via drop assay experiments described in our previous study [8]. In brief, recombinant pTR vectors harboring *copA*-like genes were first transformed into *E. coli* JW0473-3 (Δ*copA*) using a CaCl_2_-based chemical transformation method. The transformed cells were then incubated at 37 °C in the liquid LB medium supplied with 100 mg/ml ampicillin and 50 mg/ml kanamycin overnight. Cells were harvested by centrifugation, and then re-suspended in water to obtain an OD_600_ of 1.0. A gradient dilution down to 1E−4 was performed. A total of 3 μl dilution was inoculated onto LB plates containing different concentrations of Cu (1, 2, 3, and 4 mM Cu^2+^, as CuSO_4_·5H_2_O). The minimum inhibitory concentration (MIC) of the sensitive strains harboring recombinant pTR vectors was determined.

### Growth test

Cu-sensitive strains harboring recombinant or empty pTR vectors were incubated in a liquid LB medium overnight, and the initial OD_600_ was adjusted to 0.1. The growth curves of the recombinants (five *copA*-like genes and *LpcopA*) and the negative control were measured by incubating in the liquid LB medium with 2 mM CuSO_4_ at 37 °C. The concentration of each culture was measured by a BioPhotometer (Eppendorf, Hamburg, Germany) at a 30-min interval for 8 h.

### Biomass and metal sorption determination

Cu-sensitive strains harboring the recombinant or empty pTR plasmids were harvested for metal content determination using inductively coupled plasma mass spectrometry (ICP-MS; Thermo Fisher Scientific, Waltham, MA). *E. coli* strains were incubated in the liquid LB medium overnight, and the initial OD_600_ value was adjusted to 0.02. After another 8 h of incubation in the liquid LB medium with 1 mM CuSO_4_ at 37 °C, cells were collected by centrifugation at 4000 *g*. Cell pellets were rinsed with ultra-pure water and subsequently dried, weighed, and digested using 8 ml of 65% HNO_3_ and 2 ml of 70% HClO_4_. The digested mixture was dissolved in 50 ml Millipore-filtered water, and then the metal content was measured using ICP-OES (Optima 7000 DV, Perkin Elmer, MA). Certified reference material laver (GWB10023 certified by the Institute of Geophysical and Geochemical Exploration, China) was used as standard reference material for determining Cu concentration.

### Statistical analysis

All comparisons were subjected to an analysis of variance (ANOVA) using SAS (v9.4; SAS Institute, Cary, NC) general linear model (GLM). Mean separation was conducted using the Fisher’s LSD test with *P* < 0.05 considered as significant.

## Code availability

The procedure developed to mine the natural diversity of novel CopA proteins from eDNA is available at https://github.com/ebg-lab/CopA and BioCode at https://ngdc.cncb.ac.cn/biocode/tools/BT007306.

## Data availability

The 175 novel sequences have been deposited in the Genome Warehouse [55] at the National Genomics Data Center, Beijing Institute of Genomics, Chinese Academy of Sciences / China National Center for Bioinformation (GWH: GWHBISN00000000), which are publicly accessible at https://ngdc.cncb.ac.cn/gwh. These sequences have also been deposited in the GeneBank from NCBI (GeneBank: ON553002–ON553176), and can also be downloaded from Table S3.

## Competing interests

The authors declare no competing financial interests.

## CRediT authorship contribution statement

**Wenjun Li:** Validation, Formal analysis, Writing – original draft, Visualization. **Likun Wang:** Formal analysis, Writing – original draft, Writing – review & editing, Visualization. **Xiaofang Li:** Conceptualization, Visualization, Funding acquisition, Methodology, Project administration. **Xin Zheng:** Methodology, Writing – review & editing. **Michael F. Cohen:** Writing – review & editing. **Yong-Xin Liu:** Software, Visualization, Data curation, Writing – review & editing. All authors have read and approved the final manuscript.

## Supplementary material

Supplementary File S1 **A protocol for detecting CopA of high confidence from metagenomes**

Supplementary Figure S1 **Taxonomy of novel *copA* genes A.** Taxonomy of novel *copA* genes in Cladogram. The taxonomy of *copA* sequences was classified by Kraken2 based on NCBI database. The colored background represents Phylum. The labels are Family. Only the sequences having the known family are shown in this figure. **B.** Overlapping genus of known and novel *copA*. **C.** Overlapping species of known and novel copA. The taxonomy of 175 novel *copA* genes are listed in Table S3.

Supplementary Figure S2 **Experimental procedures for mining the natural diversity of CopA**

Supplementary Table S1 **Basic information of reported CopA from the fourteen bacterial species**

Supplementary Table S2 **Assemblage and blast results of the 87 metagenomic datasets**

Supplementary Table S3 **Taxonomy of 175 novel *copA* genes by Kraken2 classification**
