# Supplementary material for: Sequence-based Functional Metagenomics Reveals Novel Natural Diversity of Functional CopA in Environmental Microbiomes
Source: Genomics Proteomics Bioinformatics. 2022 Sep 8;21(6):1182–94. doi: 10.1016/j.gpb.2022.08.006 (PMC11082258; doi:10.1016/j.gpb.2022.08.006)

# File S1 A protocol for detecting CopA of high confidence from metagenomes

## 1 Preparation of local CopA database

1.1 The local CopA database comprises known CopA whose functions have been tested with experimental evidence or crystal structure resolved. Some were from the copper-resistant strain whose genomes have been fully sequenced.

1.2 The sequences were retrieved from the Uniprot database (<https://www.uniprot.org/>). Specifically, go to the official website, and search against the online database with the entry of ‘copper resistance copA’. The returned items were checked one by one, in terms of their annotation, experimental evidence, and sequence features.


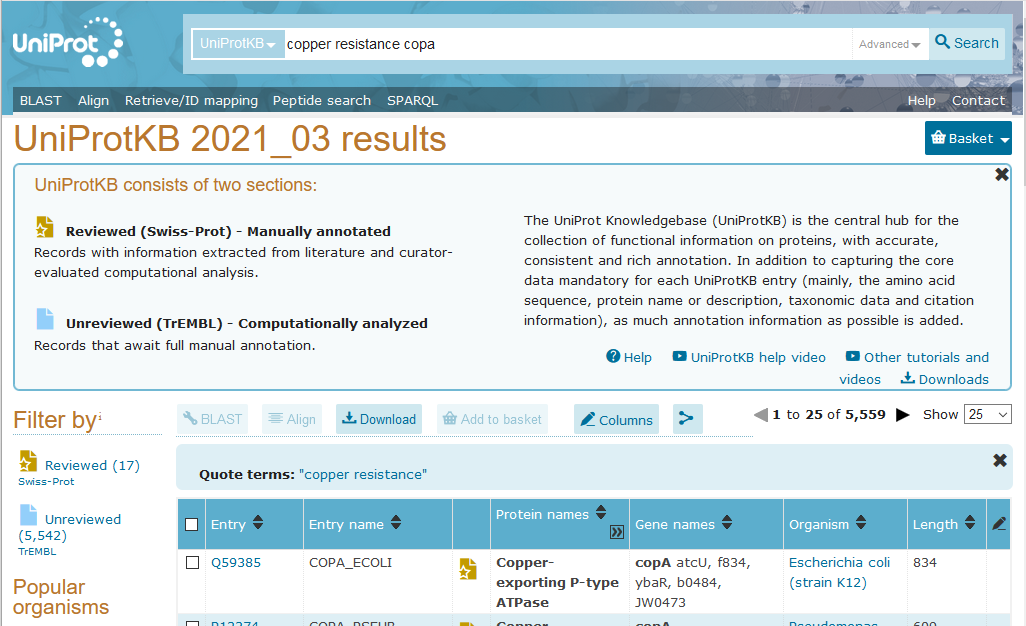


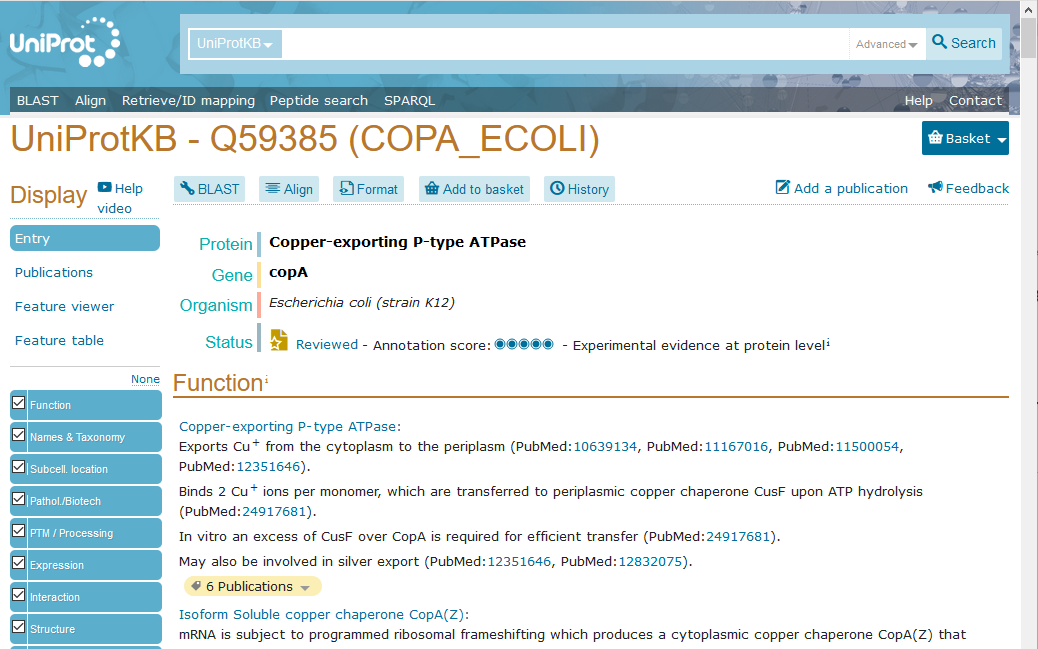


1.3 Known CopA were also searched in the literature where copper-resistant bacteria were reported and their genomes were available for annotation in National Center for Biotechnology Information (NCBI)(https://www.ncbi.nlm.nih.gov/genome/).


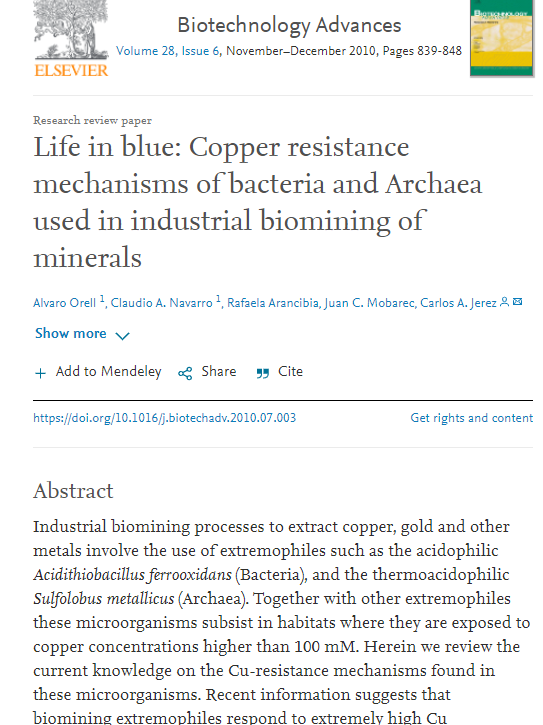


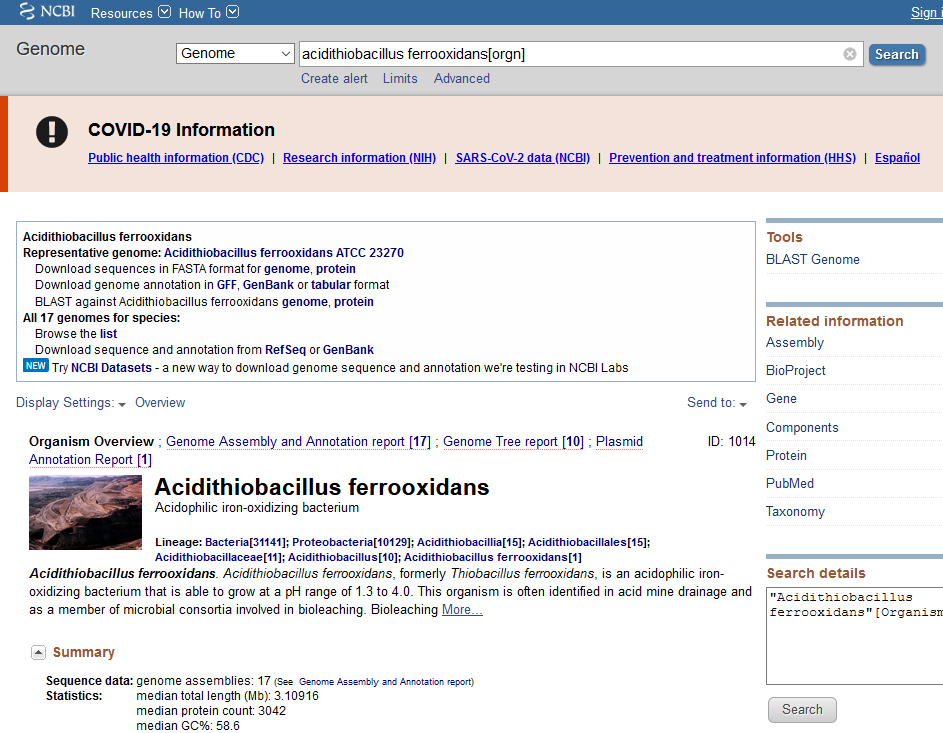


1.4 Finally, a local database named ‘CopA_reviewed.fasta’ was obtained.

1.5 Phylogenetic analysis was performed to test their evolutionary relationship.


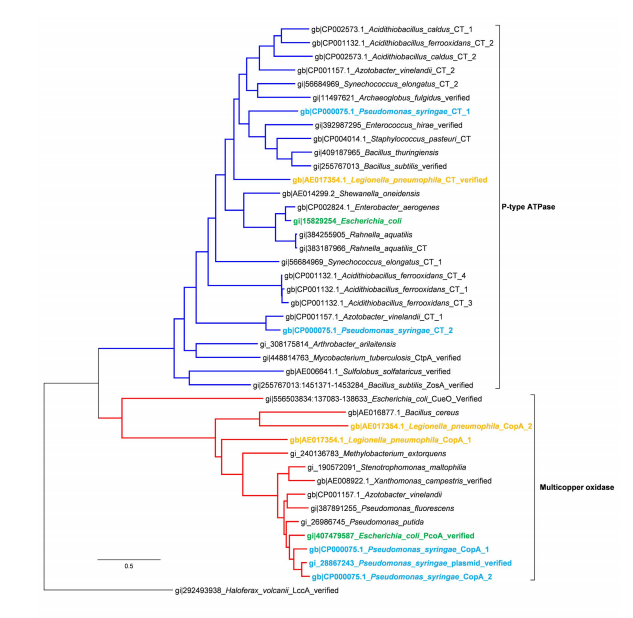


1.6 Further evolution trace analysis was done to see their length, conservative regions, and key motifs for assessing novel CopA sequences.


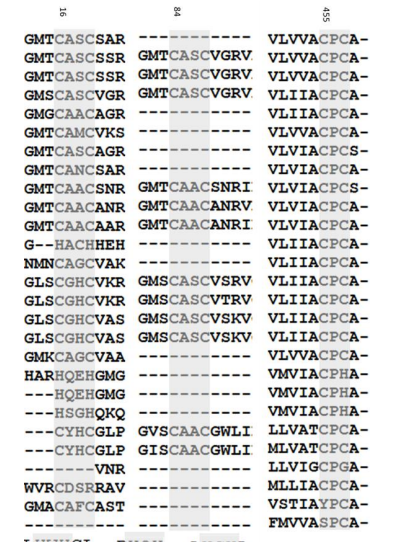


## 2 Preparation of global metagenomes for local BLAST

2.1 Download data from a public resource for the automatic phylogenetic and functional analysis of metagenomes (MG-RAST) (http://www.mg-rast.org/). Some metagenomes were downloaded as raw sequencing data, and some were processed protein assemblages.


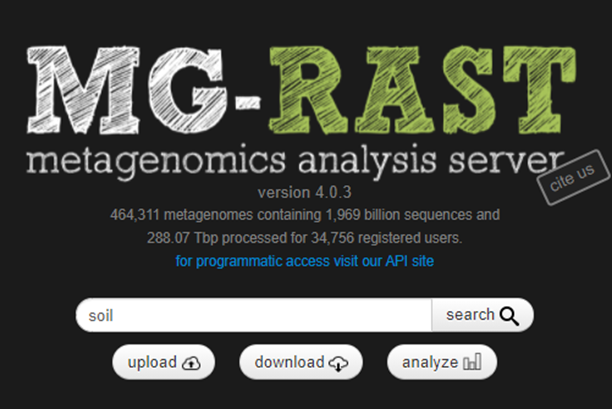


2.2 Metadata of the metagenomes including the location, data size, *etc.* were compiled manually for reference.


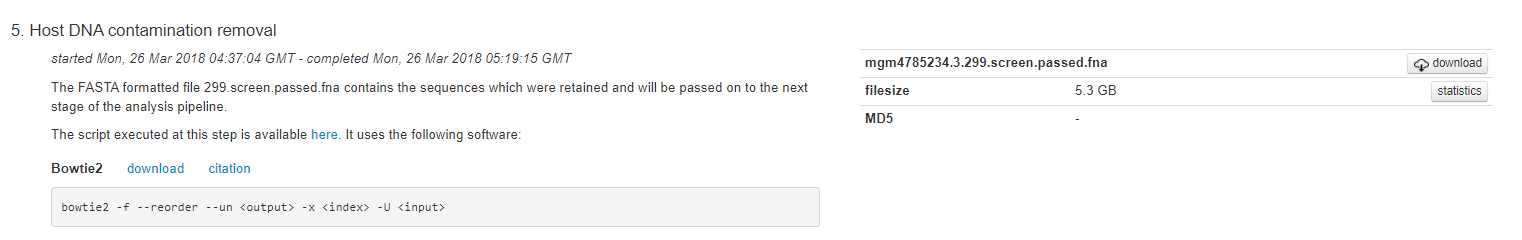


2.3 Assembly of raw data using metaWRAP.

2.3.1 Install metaWRAP from <https://github.com/bxlab/metaWRAP> v=1.2.12.3.2 Install miniconda2 by

wgethttps://repo.continuum.io/miniconda/Miniconda2-latest-Linux-x86_64.sh

bash Miniconda2-latest-Linux-x86_64.sh

2.3.3 Install metaWRAP in the conda environment

# Note: the order is important

conda config --add channels defaults

conda config --add channels conda-forge

conda config --add channels bioconda

conda config --add channels ursky

conda install -y -c ursky metawrap-mg

# Note: may take a while

# To fix the CONCOCT endless warning messages in metaWRAP=1.2, run

conda install -y blas=2.5=mkl

2.3.4 Set up the conda environment

conda create -y -n metawrap-env python=2.7

conda activate metawrap-env

# Note: the order is important

conda config --add channels defaults

conda config --add channels conda-forge

conda config --add channels bioconda

conda config --add channels ursky

conda install -y -c ursky metawrap-mg

# Note: may take a while

# To fix the CONCOCT endless warning messages in metaWRAP = 1.2, run

conda install -y blas=2.5=mkl

2.3.5 Download the database to local


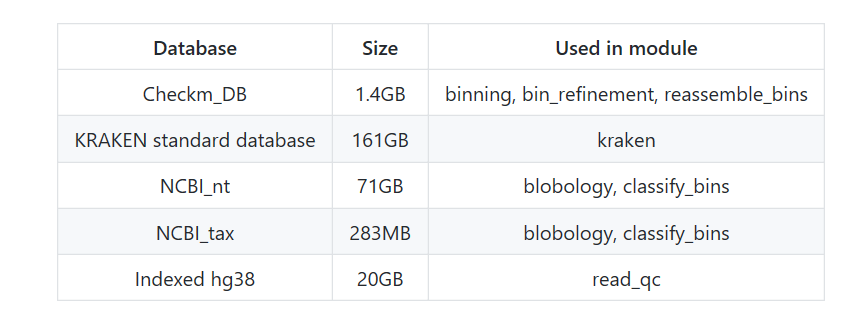


mkdir MY_CHECKM_FOLDER

checkm data setRoot

# CheckM will prompt to choose your storage location... Give it the path to the folder you just made.

#Now manually download the database @ https://github.com/bxlab/metaWRAP/blob/master/installation/database_installation.md
cd MY_CHECKM_FOLDER

wget;https://data.ace.uq.edu.au/public/CheckM_databases/checkm_data_2015_01_16.tar.gz

tar -xvf *.tar.gz

rm *.gz

2.3.6 Assemble the raw data

MataWRAP -h


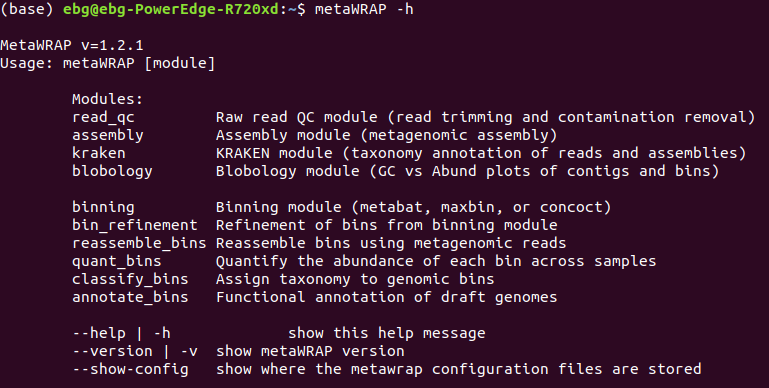


metawrap assembly -h


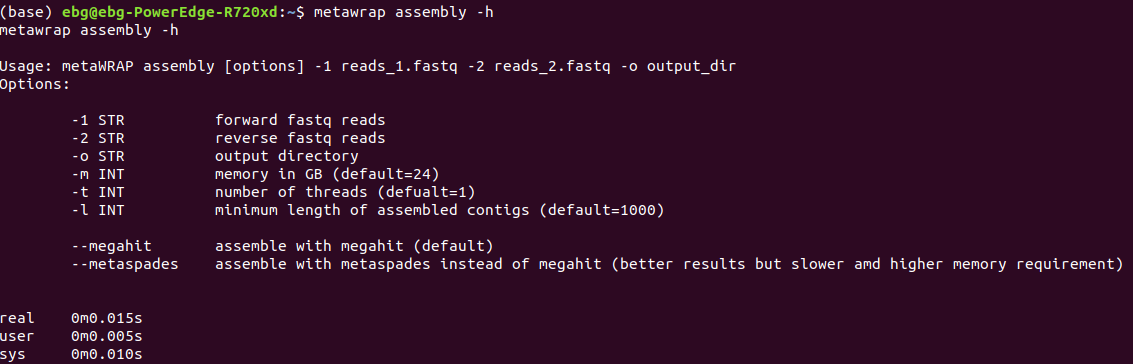


$ metawrap assembly -1 data_1.fasta -2 data_2.fasta -o output _dir

##“data.fasta” is the dataset，“data_1.fasta” is the forward sequences，“data_2.fasta” is the reverse sequences.

# and you can view the output files here


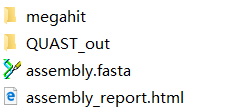


## 3 Local BLAST for detection of potential CopA

3.1 Download software blast 2.9.0 at ftp://ftp.ncbi.nlm.nih.gov/blast/executables/blast+/LATEST/. Set up the user environmental variables. Create a new variable named BLASTDB in the user variable, and the value of the variable is C：\Blast\db. Add variable value C:\Blast\bin under the system variable “Path”.

3.2 #Format the database

makeblastdb -in CopA_reviewed.fasta -dbtype prot -title " CopA_uniprot_reviewed" -out NR

#Use NR as the local database, and assembly. fasta as subject database.

blastx.exe -db NR -query assembly.fasta -out assembly.out -evalue 0.000001 -max_target_seqs 5 -num_threads 4 -outfmt6

blastp.exe -db NR -query dirp.fasta -out dirp.out -evalue 0.000001 -max_target_seqs 5 -num_threads 4 -outfmt 6

## A file with .out will be obtained.

3.3 Check the sequence quality


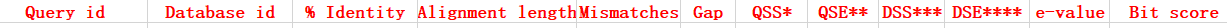


Open .out file in this excel table, and filter the sequences with proper similarity and bit score.

3.4 Manually curate the sequences of high quality, and retrieve the Open Reading Frames (ORFs) from the sequences through online ORFfinder (https://www.ncbi.nlm.nih.gov/orffinder/) and BLAST.


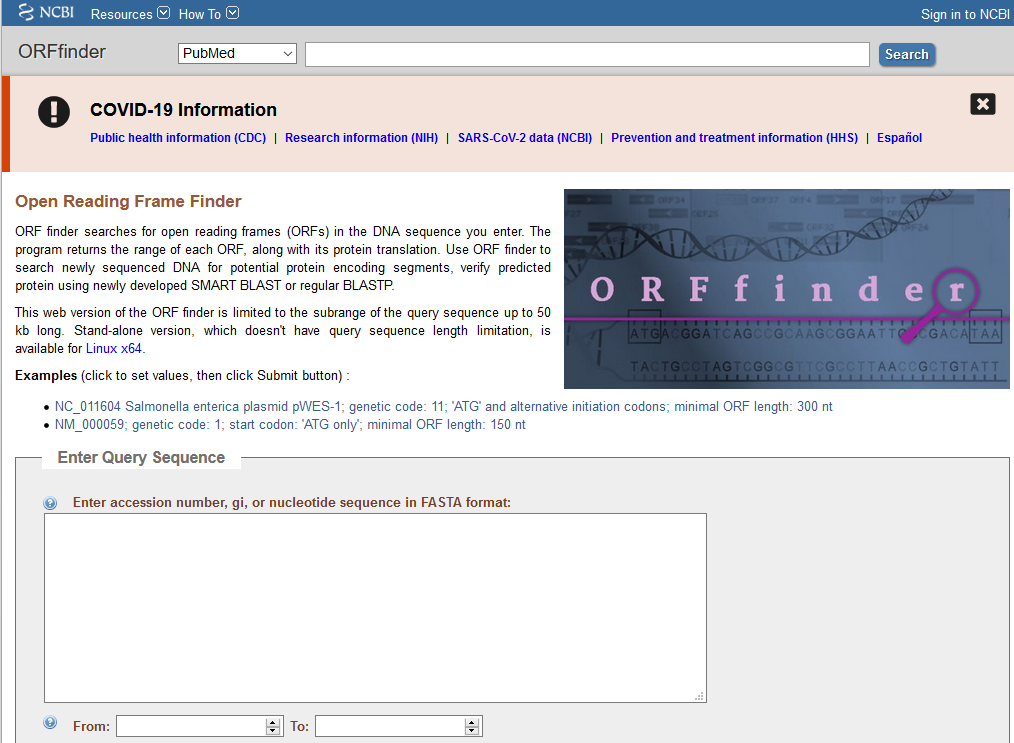


3.5 Collect the candidate *copA* genes and formed a new fasta file.


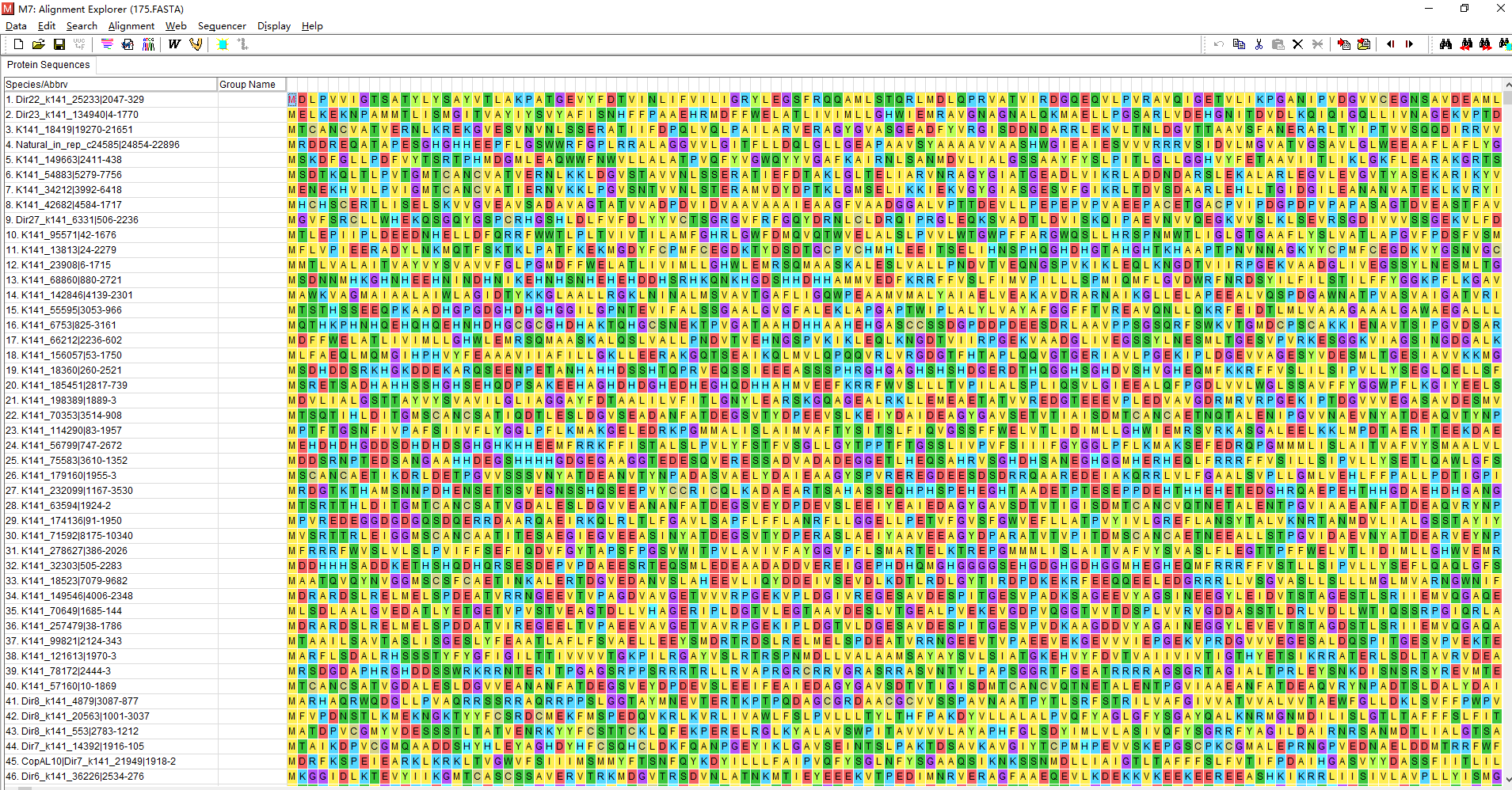


## 4 Evolutionary trace analysis of known CopA


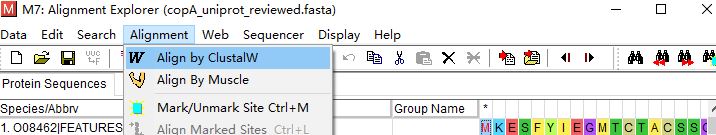


①


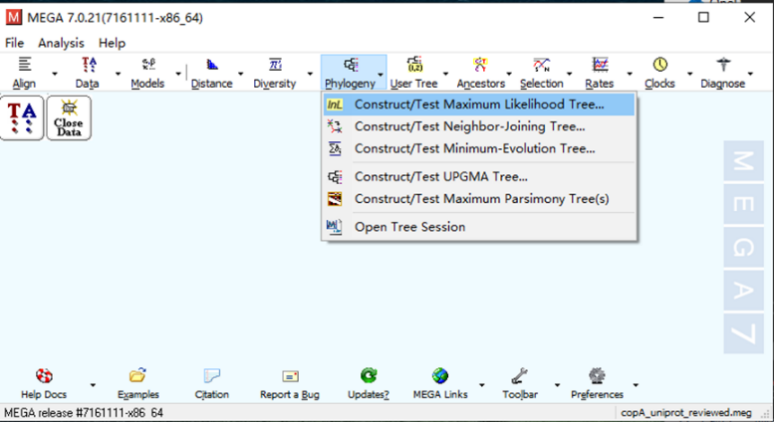


②


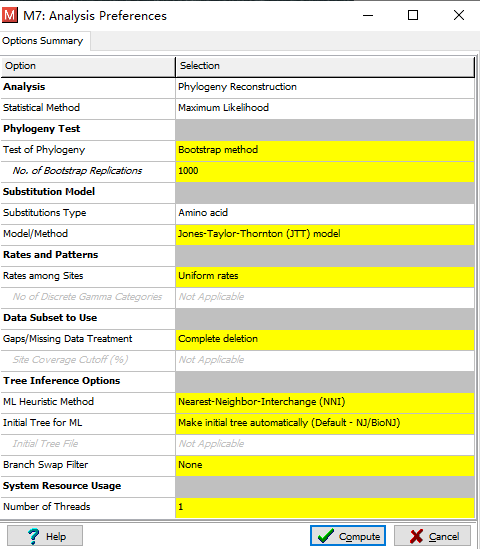


③

## 5 Bioinformatic evaluation of potential CopA

5.1 Candidate ORFs with length ranging from 500 to 900 amino acids were kept and subjected to the prediction of transmembrane helices using the TMHMM (http://www.cbs.dtu.dk/services/TMHMM-2.0/) online analysis platform.


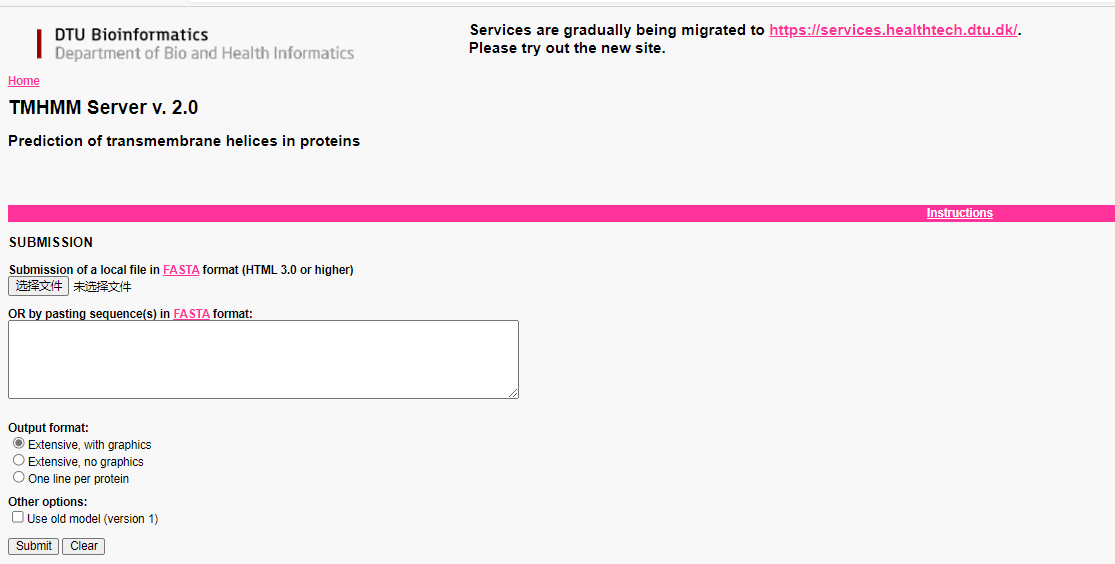


5.2 Functional domains were then predicted using Pfam (http://pfam.xfam.org/) with an *E* of ≤ 1E−6, and sequences without metal transporting domains were eliminated.


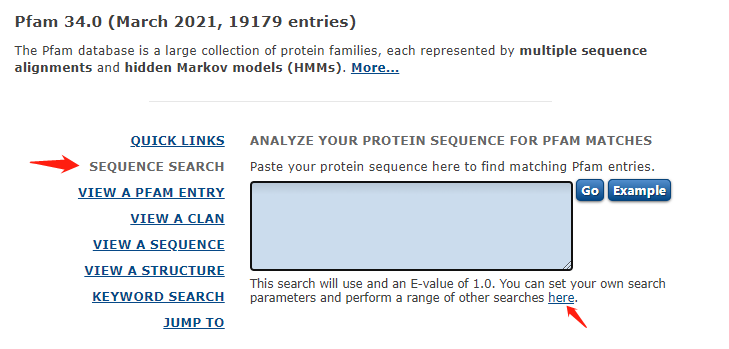


##

## 6 Phylogenetic analysis of candidate CopA

Same as evolutionary trace analysis of known CopA.

## 7 Functional genomic verification of candidate CopA (details can be found in the main text)

7.1 Chemical sysnthesis

Gene sequence optimization in order to make sure the expression of candidate *copA*. This is the key procedure of this step.

7.2 Preparation of host, positive control, and minimum inhibitory concentration (MIC) test

Synthesized genes were ligated with the PTR vector (with Kana and Amp resistance) individually, and the vectors were transformed into *copA* defective strain JW-0473-4 to verify whether the genes can restore the copper resistance of the defective strain.

7.3 Drop assay and growth test

Experimental tests on the growth of the transformed strain by growing solid LB medium with Cd supplied.


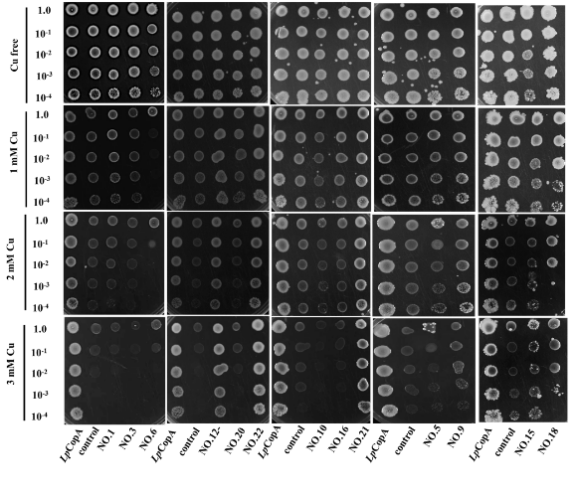


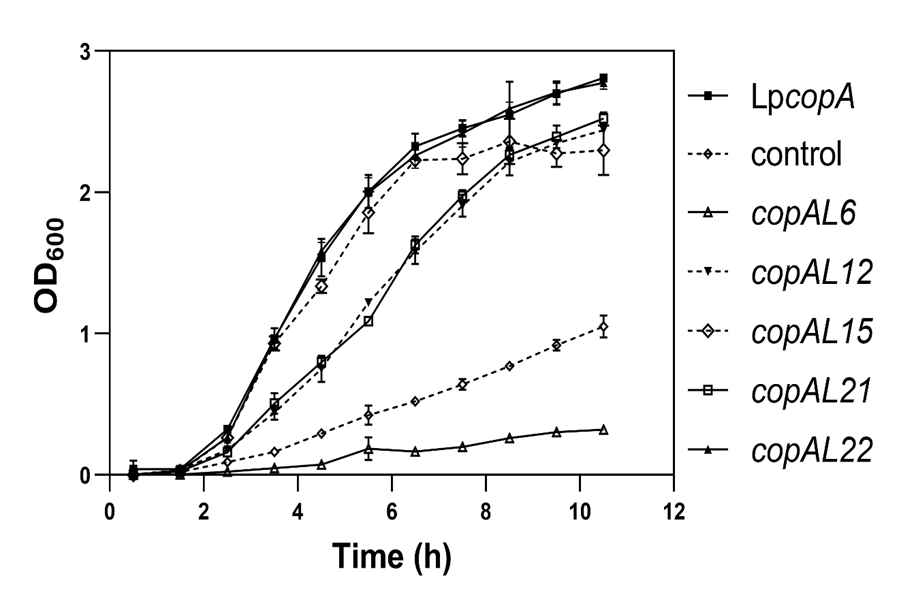

Supplement: Supplementary File S1 — A protocol for detecting CopA of high confidence from metagenomes [file mmc1.docx]
