# Supplementary figures and images for: Sequence-based Functional Metagenomics Reveals Novel Natural Diversity of Functional CopA in Environmental Microbiomes
Source: Genomics Proteomics Bioinformatics. 2022 Sep 8;21(6):1182–94. doi: 10.1016/j.gpb.2022.08.006 (PMC11082258; doi:10.1016/j.gpb.2022.08.006)

**A**

Proteobacteria  
 Actinobacteria  
 Bacteroidetes  
 Firmicutes  
 Euryarchaeota

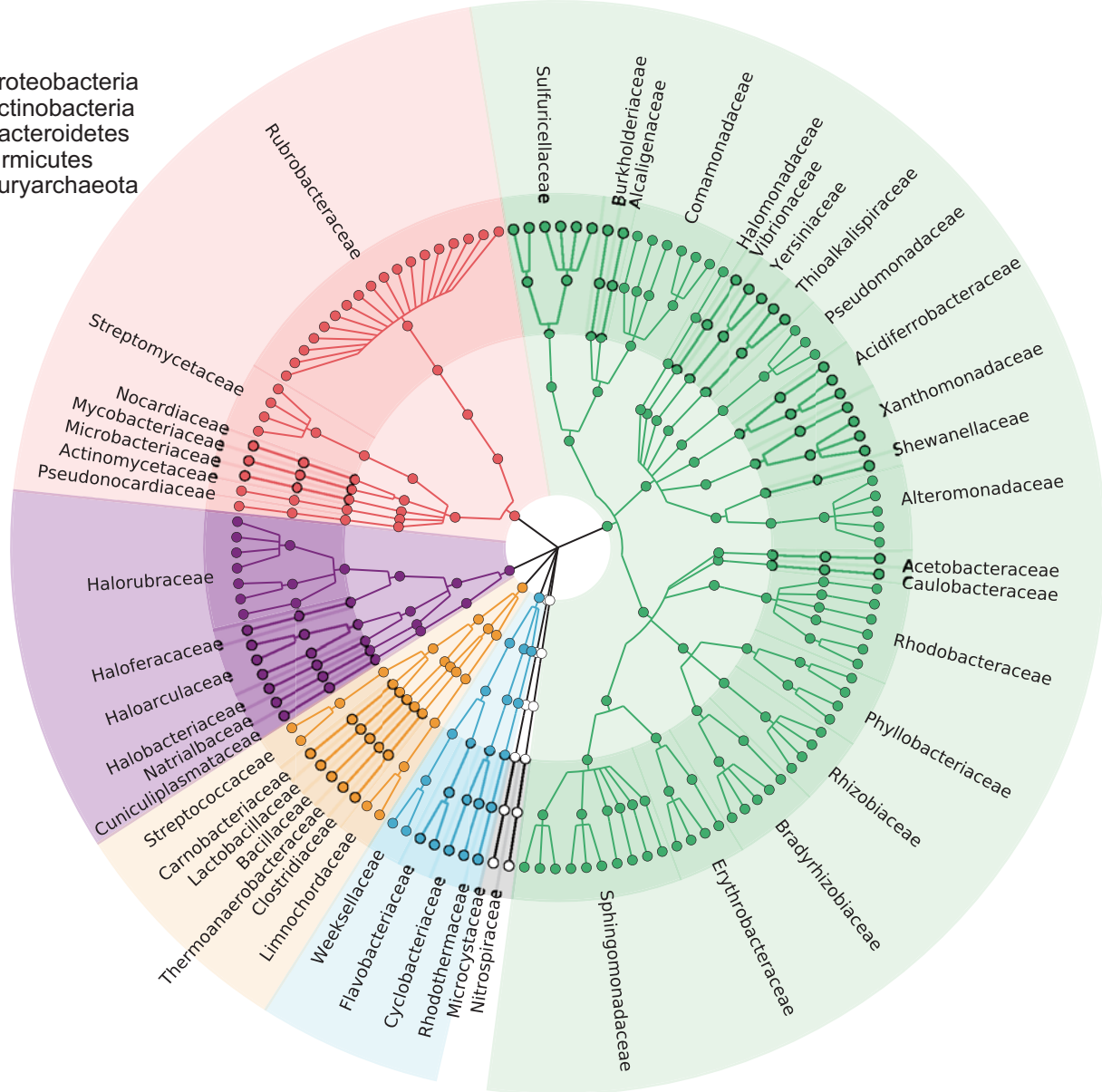**B**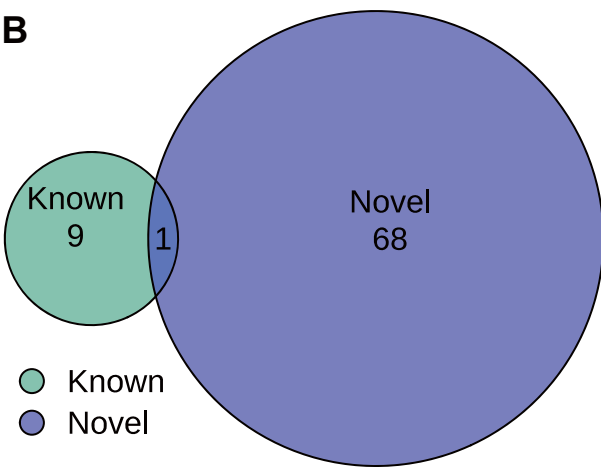**C**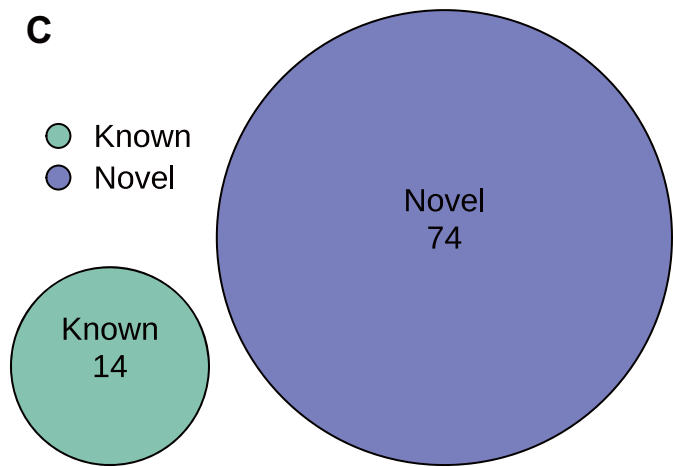

Supplement: Supplementary Figure S1 — Taxonomy of novel copA genes A. Taxonomy of novel copA genes in Cladogram. The taxonomy of copA sequences was classified by Kraken2 based on NCBI database. The colored background represents Phylum. The labels are Family. Only the sequences having the known family are shown in this figure. B. Overlapping genus of known and novel copA. C. Overlapping species of known and novel copA. The taxonomy of 175 novel copA genes are listed in Table S3. [file mmc2.pdf]

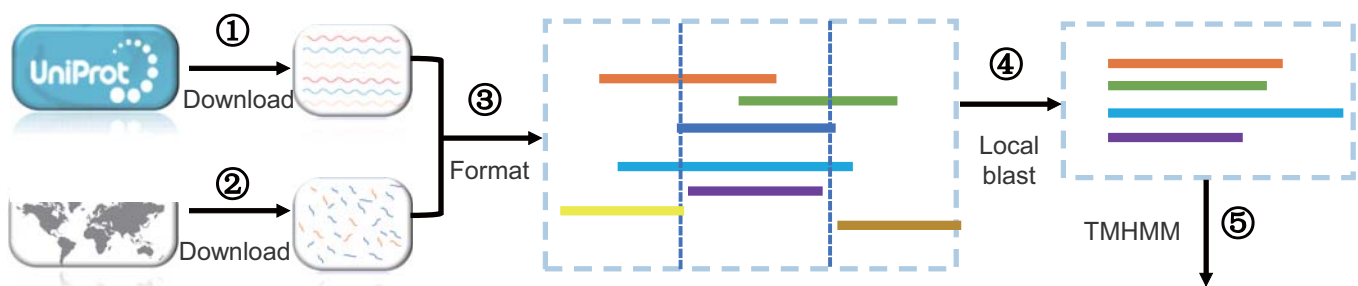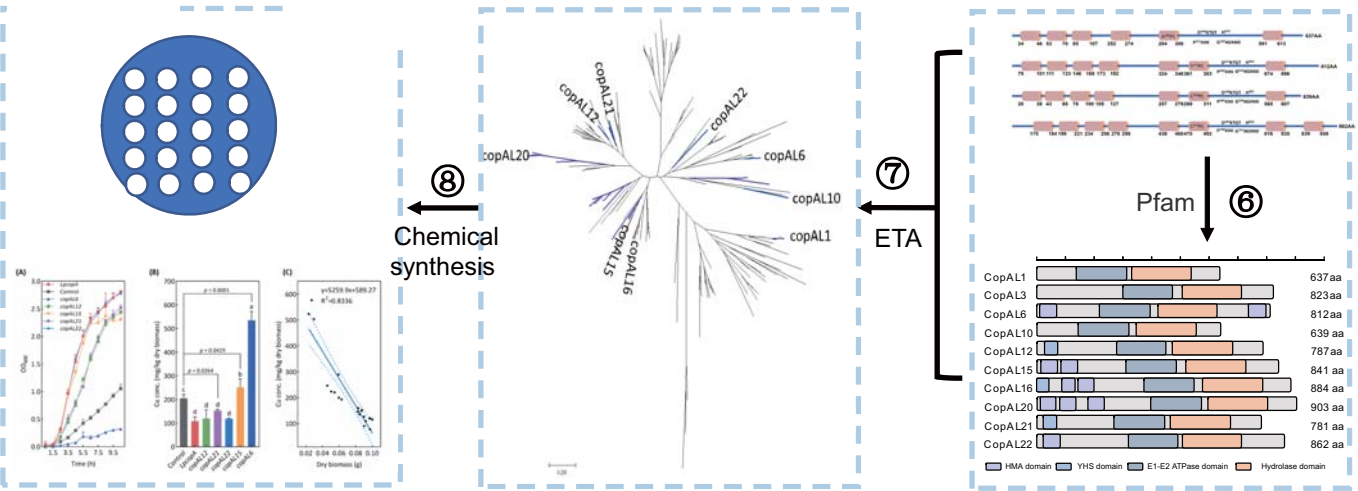

Supplement: Supplementary Figure S2 — Experimental procedures for mining the natural diversity of CopA [file mmc3.pdf]
